# Topical and Systemic Formulation Options for Cutaneous T Cell Lymphomas

**DOI:** 10.3390/pharmaceutics13020200

**Published:** 2021-02-02

**Authors:** Taku Fujimura, Ryo Amagai, Yumi Kambayashi, Setsuya Aiba

**Affiliations:** Department of Dermatology, Tohoku University Graduate School of Medicine, Sendai 980-8574, Japan; amagai@derma.med.tohoku.ac.jp (R.A.); yumi1001@hosp.tohoku.ac.jp (Y.K.); saiba@med.tohoku.ac.jp (S.A.)

**Keywords:** CTCL, topical formulation, bexarotene, targeted therapy, interferon, HDAC inhibitors

## Abstract

Although various anti-cutaneous T-cell lymphoma (CTCL) therapies are available for clinical use, appropriate chemotherapy lines for the treatment of CTCLs have yet to be established. Therefore, to date, various clinical trials for the treatment of advanced CTCLs are ongoing. In this review, we evaluate the therapeutic options that are available in clinical practice for treatment of early- and advanced-stage CTCLs (targeted therapies, histone deacetylase (HDAC) inhibitors, retinoids, interferons, cytotoxic drugs, etc.). We also examine clinical trials of novel regimens for the treatment of CTCLs.

## 1. Introduction

Most cutaneous T-cell lymphomas (CTCLs), such as mycosis fungoides (MF), start as an indolent disease that progresses slowly before advancing to skin tumors followed by lymph node and visceral involvement [1,2]. The overall incidence of CTCL is 10.2 per million persons, most of which are MF cases that present an indolent clinical course [3]. The overall survival (OS) of MF/Sezary syndrome (SS) depends on the clinical stage. MF patients with stage IA show a median survival of 35.5 years and a disease-specific survival (DSS) of 90% at 20 years. Patients with stage IIA also have good prognosis: the median survival is 15.8 years and the DSS is 60% at 20 years [3]. However, patients with stage IIB have a median survival of 4.7 years and a DSS of 56% at 5 years and 29% at 20 years. Since the median survival and DSS at or beyond stage IIIA is worse than that of stage IIB, it is important to evaluate systemic therapies for advanced stage (beyond stage IIB) MF.

As a result of the disease’s immunological background, early-stage MF resembles skin inflammatory disorders such as atopic dermatitis (AD) [4,5,6]; as a result, topical formulation therapies play important roles in treating early-stage MF. In contrast, advanced-stage MF is treated using various systemic therapies. Although the National Comprehensive Cancer Network (NCCN) guidelines for primary cutaneous lymphomas suggest three categories of systemic therapies as options for the treatment of advanced MF/SS, unlike the guidelines for solid tumors, the recommended first-line treatments for each category have yet to be established [1]. Indeed, category A systemic therapy (SYST-CAT A) contains eight regimens, whereas SYST-CAT B contains four regimens and the category for large cell transformation (LCT) contains five regimens, suggesting that the selection of first-line therapy is up to each dermatologist or hemato-oncologist [1]. Therefore, it is important to understand the characteristics of each regimen for advanced MF/SS. In this review, we evaluate the therapeutic options for early- and advanced-stage CTCLs available in clinical practice in order to encourage the selection of appropriate chemotherapy regimens for each therapy line.

## 2. Topical Formulation Options for CTCLs

### 2.1. Topical Steroids

Topical steroids have been used classically and widely for the treatment of early-stage MF [7,8]. The overall response rates (ORRs) are 94% for Stage I disease and 82% for Stage II disease [8]. Various mechanisms of anti-CTCL effects of topical steroids have been reported, including the induction of apoptosis of CTCL cells and downregulation of nuclear factor-ΚB and the NF-KB activation protein, with associated decreases in cytokine, chemokine, and adhesion molecule production [7]. More recently, Furudate et al. reported that the immunological background (e.g., stromal factors, chemokine profiles) of early-stage MF is similar to that of AD [4], suggesting the utility of topical steroids for the treatment of early-stage MF.

### 2.2. Topical Retinoids

Bexarotene gel is a topical drug that has been approved by the US Food and Drug Administration (FDA) for the treatment of early-stage MF [7,9]. The ORR of topical bexarotene gel is 63%, including a clinical complete response (CR) of 21% after a median treatment interval of 20 weeks [9]. The major adverse event (AE) associated with bexarotene gel is irritation (87%), which is increased with the number of times of gel exposure [9]. The incidence of severe AEs (SAEs) was 11.9% (8/67), but the SAEs were not treatment related [9]. Overall, the safety profile of bexarotene gel indicates that topical bexarotene therapy is well tolerated for routine use with twice-daily dosing [9]. Several preclinical studies have suggested the anti-CTCL mechanisms of bexarotene [10,11,12,13]. For example, bexarotene increases integrin β7 expression by CTCL cells, resulting in the induction of growth arrest and apoptosis [11]. In addition, bexarotene reduces the production of C–C motif chemokine (CCL)22 from tumor-associated macrophages (TAMs), thereby suppressing the recruitment of CTCL cells and regulatory T cells in the lesional skin of MF [12]. Bexarotene also decreases C-C chemokine receptor type 4 (CCR4) expression by CTCL cells, suppressing chemotaxis in SS [13]. Since both CTCL cells and TAMs are distributed in the superficial dermis in early-stage MF [4], topical bexarotene may be useful only for the treatment of early-stage MF.

Tazarotene 0.1% gel, another topical retinoid, also is used for the treatment of early-stage MF [14]. The efficacy of tazarotene 0.1% gel was 58% (11/19) in an intent-to-treat analysis [14]. Although all patients developed mild to moderate skin irritation, the toxicity of tazarotene gel is limited to a local reaction.

### 2.3. Topical Nitrogen Mustard (Mechlorethamine Hydrochloride)

In 2013, nitrogen mustard (NM), an alkylating agent, was approved by the FDA for the treatment of early-stage MF (Stage IA/IB) with previous skin-direct therapy [15]. The ORRs of mechlorethamine hydrochloride gel are 46.9% by the Modified Severity-Weighted Assessment Tool (mSWAT), and 58.6% by the Composite Assessment of Index Lesion Severity-Weighted Assessment Tool [15]. A corresponding multicenter trial observed no drug-related SAEs among 130 patients with early-stage MF, but 20.3% of enrolled patients discontinued treatment because of drug-related skin irritation [15]. More recently, the clinical use of NM gel in 298 patients with early-stage MF has been reported, suggesting that about half of the subjects in this cohort used concomitant therapies such as corticosteroid (24%), phototherapy (12%), or systemic retinoid (10%) [16]. Notably, NM might induce an anti-CTCL immune response, along with DNA damage in lymphoma cells [17]. Therefore, in clinical practice, NM is administered as a combination therapy together with other local or systemic therapies.

### 2.4. Topical Carmustine (Bis-Chloroethyl-Nitrosourea, BCNU)

BCNU is a nitrosourea alkylating agent that has been used for the treatment of early-stage MF [18,19]. A previous report on the treatment of 87 cases of early-stage MF with BCNU found an ORR of 98% for Stage-IA patients (10% skin involved), including 86% CR and 12% PR; and 84% for patients with Stage-IB (≥10% skin involved) disease, including 47% with CR and 37% with PR [18]. In addition, long-term analysis of 188 subjects with early-stage MF treated with BCNU revealed that 91% of patients with Stage-IA disease and 62% of patients with Stage-IB disease continued to use topical BCNU at 36 months after the initiation of treatment. The major AEs with BCNU were irritation with burning sensation and contact dermatitis [18]. Mild leukopenia occurred in 3.7% of BCNU-treated patients, but there were no instances of treatment failure caused by leukopenia [18]. As with NM, BCNU induces the apoptosis of lymphoma cells by interstrand cross-linking of DNA [19,20], suggesting that BCNU may induce an anti-CTCL immune response.

### 2.5. Topical 5% Imiquimod (IQM)

Imiquimod (IQM) is an immunomodulatory, small-molecule compound in the imidazoquinoline family; this compound induces antitumor effects through Toll-like receptor 7 (TLR7). As a TLR7 agonist, IQM stimulates the cells of innate immunity, such as plasmacytoid dendritic cells (DCs) and TAMs, to produce pro-inflammatory cytokines (e.g., IFN-α and TNF-α) [21,22]. Since a substantial number of TAMs surrounded by CTCL cells are distributed in the lesional skin of MF [4], topical 5% IQM may suppress the progression of MF by polarizing TAMs. Indeed, the ORR of topical 5% IQM for the treatment of MF (Stage IA-IIB) was 80% (16/20), including 45% CR and 35% PR [23]. The major AEs with topical 5% IQM were limited to the local lesion [23], although flu-like symptoms were noted in another case report [24]. Notably, topical 5% IQM is effective even against advanced-stage MF (Stage IIB). This activity might be explained by previous preclinical studies that focused on TAMs [12,22,25]. Since serum CCL22 levels represent disease activity in patients with early- and advanced-stage MF [12,25], and the production of CCL22 from TAMs is suppressed by topical 5% IQM in vivo (in a B16F10 mouse melanoma model) [22], the administration of topical 5% IQM may decrease serum CCL22 levels, leading to the suppression of tumor progression in patients with advanced-stage MF. Since the case series of advanced-stage MF treated with topical 5% IQM are limited, further case series will be needed to prove the efficacy of IQM.

### 2.6. Future Perspectives

As described above, there are several topical formulation options for the treatment of early CTCL (Table 1). Topical formulations are even useful for advanced CTCL, although in most patients, they need to be combined with systemic therapies. Since local therapies such as retinoids, nitrogen mustard, and bexarotene directly kill CTCL cells at a tumor sites to provide tumor antigens, these local therapies might enhance systemic immunomodulatory therapies. For example, as we described above, since topical bexarotene [12] or imiquimod [22] reduces the production of CCL22, leading to suppression of the recruitment of CTCL cells and regulatory T cells in the lesional skin of MF, either combined with surface molecular-targeted therapy described in next section might be suitable to complete the reduction of CTCL. Topical NM or BCNU directly kill CTCL cells [15,18], which might be suitable for combination with systemic immunotherapy such as mogamulizumab [26]. The efficacy of such combined therapies should be evaluated in the future.

## 3. Systemic Treatment Options for Advanced CTCLs

### 3.1. Surface Molecular-targeted Therapy for Advanced CTCLs

Among surface molecular-targeted therapies, mogamulizumab, brentuximab vedotin, denileukin diftitox, alemtuzumab, and pembrolizumab are recommended by the 2020 NCCN guidelines for the treatment of primary cutaneous lymphomas [1] (Table 2).

#### 3.1.1. Mogamulizumab

Mogamulizumab is a humanized anti-CCR4 antibody that shows cytotoxicity against CCR4+ lymphoma cells via antibody-dependent cell-mediated cytotoxicity (ADCC) in CTCL patients. A recent clinical trial revealed that mogamulizumab is one of the most effective systemic therapies for relapsed CTCL [26]; therefore, this antibody is recommended by the NCCN as an effective systemic therapy for MF (Stage IIB) and SS [1], although the chemotherapy lines for the treatment of advanced CTCL are still under discussion [2]. The median progress-free survival (PFS) of mogamulizumab for relapsed CTCL was 7.7 months (95% confidence interval (CI): 5.7–10.3 months), which is superior to that of vorinostat (3.1 months: 95% CI: 2.9–4.1 months) (hazard ratio (HR) 0.53; 95% CI 0.41–0.69; stratified log-rank *p* < 0.0001) [26]. The ORR was 28% (52/186) in the mogamulizumab cohort, compared to 5% (9/186) in the vorinostat cohort. Overall median times to response were 3.3 months (IQR 2.0–6.4) in the mogamulizumab group and 5.1 months (2.9–8.5) in the vorinostat group. The most common treatment-related AEs in the mogamulizumab group were infusion reaction (32%), drug eruption (20%), diarrhea (23%), and fatigue (22%) [25]. In addition, the major SAEs by mogamulizumab were pyrexia (4%) and cellulitis (3%), suggesting that mogamulizumab is a well-tolerated systemic therapy for advanced CTCL [26].

Since the ORR of mogamulizumab monotherapy for relapsed CTCL is 28%, several mogamulizumab-based combination therapies have been reported recently [27,28,29,30]. For example, the combined administration of etoposide and mogamulizumab was shown to be useful for the treatment of mogamulizumab-resistant MF [28,29]. Notably, using an in vivo model (EL4 mouse lymphoma model), the intraperitoneal administration of etoposide significantly increased the expression in implanted tumors of mRNAs encoding CCL17, CXCL5, and CXCL10 [28]. Those researchers concluded that CCR4+ CTCL cells, as well as CXCR3+ effector cells and CXCR2+ monocytes, accumulate in tumor sites, leading to potentiation of the ADCC activities of mogamulizumab [28]. Other reports have suggested that mogamulizumab monotherapy may be augmented by the concurrent use of radiation therapy [27,30]. Although mogamulizumab has been approved by the FDA for the treatment of CTCL since 2018, the use of mogamulizumab in clinical practice is still limited. Further case series or clinical trials for mogamulizumab-based combination therapies are needed to confirm the efficacy of mogamulizumab.

#### 3.1.2. Brentuximab Vedotin

Brentuximab vedotin is an anti-CD30 monoclonal antibody conjugated to monomethyl auristatin E via a protease-cleavable linker; this biologic is used for the treatment of CD30+ lymphoma, including CTCL [31,32,33]. Recently, a randomized phase III multicenter trial was performed to evaluate the efficacy of brentuximab vedotin by comparing with the physician’s choice for the treatment of CD30+ CTCL [32]. The population of patients achieving an objective global response lasting at least 4 months was 56.3% (36/64) with brentuximab vedotin, compared to 12.5% (8/64) in the physician’s choice group (*p* < 0.0001) [32]. The incidence of treatment-related SAEs with brentuximab vedotin was 41%, and the most common SAEs were peripheral sensory neuropathy (5%) and fatigue (5%). The most common AEs of any grade were peripheral sensory neuropathy (50%), nausea (38%), fatigue (34%), and diarrhea (32%) in patients with CTCL treated with brentuximab vedotin.

In addition to the clinical trial described above, a literature review of CD30+ CTCL patients treated with brentuximab vedotin found an ORR of 64.0%, including primary cutaneous anaplastic large cell lymphoma (PC-ALCL) (for which the ORR was 100%: 7/7) and CD30+ MF (for which the ORR was 64%: 39/61). The CR rate was 100% for the PC-ALCL cohort compared to 6.6% for the MF cohort [31]. Median duration of response (DOR) for brentuximab vedotin was 15.1 months (95% CI 9.7–25.5). Notably, brentuximab vedotin is also useful for low CD30-expressing (<5% CD30 expression) MF/SS [34]. This discrepancy might be explained by the affinity of anti-CD30 Abs for immunohistochemical staining or the depletion of CD30 co-expressing CD163+ TAMs, which show immunosuppressive phenotypes in advanced CTCL. Although the profile of the most common AEs of any grade was similar to that obtained in the clinical study [32], the frequency of AEs differed between the two reports [31]. The most common AEs of any grade in the literature review were peripheral neuropathy (57.2%), fatigue (35.6%), nausea (19.5%), and diarrhea (11.7%) [31].

#### 3.1.3. Denileukin Diftitox

Denileukin diftitox is a chimeric immunotoxin consisting of a fusion of the full-length human IL-2 protein with a modified cytotoxic domain of diphtheria toxin [35]. Following the binding of this chimera to cells expressing intermediate- or high-affinity IL-2 receptor, the diphtheria toxin is released by cleavage, thereby inducing cytotoxicity in the target cells. A phase III placebo-controlled trial of denileukin diftitox for early- and advanced-stage CTCL revealed an ORR of 44% (44/100), which is a value that included a 10% CR and a 34% PR: values that were significantly superior to the corresponding numbers for placebo-treated patients (15.9% ORR consisting of 2% CR and 13.6% PR) [36]. The PFS was significantly longer in patients treated with denileukin diftitox compared to those treated with placebo (median, >2 years vs. 124 days, respectively; *p* < 0.001). The median DOR was 236 days, and the time to response was 96 days. The incidences of SAEs reported in this clinical study were 34% in the denileukin diftitox arm and 32% in the placebo arm [36], and incidences of treatment-related SAEs were 4% (4/100) and 0%, respectively. The most common treatment-related SAEs were dehydration (2%) and capillary leak syndrome (2%) [36]. Notably, Kadin et al. reported that patients with erythrodermic CTCL, including SS, might be at an increased risk of capillary leak syndrome [35]. In addition, nausea (10%), fatigue (12%), pyrexia (11%), and rigors (12%) were reported as common moderately severe treatment-related AEs [36]. In another clinical trial for denileukin diftitox retreatment of patients with relapsed CTCL, the ORR of denileukin diftitox retreatment was 40% (8/20), and median intent-to-treat PFS was 205 days [37], which was comparable to the PFS reported in the previous phase III trial [36]. In addition, the profiles of the most common AEs (nausea, fatigue, rigor) were highly similar in these two phase III trials. Together, these data indicated that denileukin diftitox is an effective and well-tolerated systemic therapy for early- and advanced-stage CTCL, even in relapsed cases, although the incidence of capillary leak should be carefully monitored.

#### 3.1.4. Pembrolizumab

Anti-programmed cell death 1 (PD-1) antibodies are in wide use for the treatment of various cancers, including both solid cancers and hematological malignancies [38,39]. Given that T-cell lymphomas, including MF and SS, involve malignant T cells [40,41], the blockade of PD-1/PD-L1 signaling could be a target for the treatment of CTCLs such as MF. Indeed, Khodadoust et al. reported a multicenter phase II study of pembrolizumab in relapsed and refractory cases of advanced MF and SS that had been heavily pretreated with a median of four prior systemic therapies, including other targeted therapies, histone deacetylase (HDAC) inhibitors, interferons, and bexarotene [40]. The ORR in this study was 37.5% (9/24) with two cases showing CR (8.3%) and seven cases showing PR (29.2%). Median DOR was not reached during a median follow-up time of 58 weeks [40]. Notably, in CTCL cohorts, treatment response did not correlate with PD-L1 expression, total mutation burden, or interferon gamma-encoding gene expression signature. The incidence of severe immune-related AEs (irAEs) was 16.7% (4/24), which is comparable to the incidence of SAEs in other types of cancer such as advanced melanoma [42]. In addition, the profiles of SAEs (e.g., pneumonia, colitis, liver dysfunction) were similar to those seen with other types of cancer [42]. Notably, since various irreversible endocrine disorders such as isolated ACTH deficiency, destructive thyroiditis, and adrenal dysfunction could develop in patients who are administered anti-PD1 Abs [38,43], oncologists should take into account the occurrence of such irreversible AEs.

#### 3.1.5. Alemtuzumab

Alemtuzumab is an anti-CD52 antibody that shows cytotoxicity against CD52+ lymphoma cells via ADCC in mature T cell and NK cell malignancies, including erythrodermic MF and SS [44]. de Masson et al. reported the results of a study involving 39 patients with CTCL (23 with SS and 16 with advanced MF) who were treated with alemtuzumab [45]. The ORR was 51.1% (20/39) with seven cases of CR (17.9%) and 13 cases of PR (33.3%). Notably, the ORR was significantly higher in patients with SS [70% (16/23)] compared to those with MF [25% (4/16)] (*p* < 0.009). The median PFS was 3.4 months (range 0.4–42 months). Twenty-four patients (62%) had a severe infectious AE, and 10 patients (26%) had a hematological toxicity. Another report described a case series of 19 heavily pretreated patients with CTCLs who subsequently were treated with alemtuzumab; the ORR was 84% (16/19), with nine cases of CR (47%) and seven cases of PR (37%) [46]. Median overall survival (OS) was 41 months, whereas median PFS was 6 months [47]. In the absence of a prospective clinical trial of this biologic, further study will be needed to assess the efficacy of alemtuzumab for the treatment of CTCL.

### 3.2. Immunomodulatory Reagents: Interferon (IFN), Bexarotene, Etoposide

Immunomodulatory reagents have been used classically for the treatment of CTCLs. Among these reagents, IFN and bexarotene recently have been investigated for their immunomodulatory effects on the CTCL tumor microenvironment [12,28,47,48,49]. These reports suggest the possible utility of combination therapies for CTCL, such as IFN-α2a in combination with psoralen with ultraviolet light A (PUVA), low-dose methotrexate, or retinoid [50,51,52], or bexarotene plus phototherapy [53,54]. Preclinical findings suggest that more immunomodulatory reagent-based combination therapies will be established in the future.

#### 3.2.1. Interferon

Both IFN-α and IFN-γ are effective and have been used classically for the treatment of CTCLs [55], although the mechanism of the induction of the anti-lymphoma response is not fully understood [47,48,49,50,51,52]. For example, both IFN-α and IFN-γ re-polarize TAMs to suppress the production of CCL22 [47], which recruits CCR4+ T cells, including lymphoma cells and regulatory T cells. Notably, recent reports also suggest that serum CCL22 levels correlate with the disease activity of MF, indicating that CCL22 could be a biomarker that reflects the response of MF to compounds such as bexarotene and mogamulizumab [12,25]. Taken together, these data suggest that IFNs may induce an anti-CTCL response by inducing changes in the chemokine profiles of TAMs in the CTCL tumor microenvironment.

The efficacy of IFN-α monotherapy and combined therapy for the treatment of CTCL has been reported over several decades [55,56,57,58]. Indeed, a phase II study to evaluate the efficacy of high-dose IFN-α2a monotherapy found an ORR of 29% (7/24), with one case of CR and six of PR [59]. In addition, IFN-α achieved a superior time-to-next-treatment (TTNT) compared to chemotherapy in all stages of MF [55]. The median TTNT for IFN-α was 8.7 months (95% CI 6.0–18.0 months), which was superior to that obtained for single or multiagent chemotherapy (3.9 months; 95% CI: 3.2–5.1 months) and HDAC inhibitors (4.5 months; 95% CI: 4.0–6.1 months) [56]. These reports suggest that IFN-α monotherapy is effective against previously treated MF and SS [56,58].

The efficacy of IFN-α has also been reported as part of a combination therapy [50,51,52]. A phase II trial evaluated the efficacy of IFN-α2a plus PUVA therapy in a study that enrolled 40 patients with early MF and 23 patients with advanced MF, including two SS patients [50]. The ORR was 80.6% (55/63), including 51 cases with CR (74.6%) and four with PR (6%). Notably, the median response duration was 32 months, and no life-threatening side effects were observed [50]. More recently, Olisova et al. reported a retrospective analysis of 22 MF patients (including 34% with early-stage MF and 66% with advanced-stage MF) who had been treated with IFN-α2b in combination with PUVA therapy [51]. The ORR was 93% (20/22), with 16 cases of CR (73%) and four of PR (20%). In addition, the 2-year PFSs were 100% in patients with early-stage MF and 82% in patients with advanced-stage MF, while the 5-year PFSs were 90% and 43%, respectively [51]. These reports suggest that IFN-α2a or IFN-α2b in combination with phototherapy is effective and well-tolerated in patients with symptomatic MF [50,51]. Not only phototherapy, but also methotrexate (MTX) and retinoids could be combined with IFN-α. Aviles et al. reported a randomized study of refractory/relapsed CTCL treated with IFN in combination with low-dose MTX (201 patients) or treated with IFN in combination with retinoid (176 patients) [52]. The overall CR rate was 80% (162/201) for the IFN plus low-dose MTX arm, and 80% (141/176) for the IFN plus retinoid arm. Moreover, the 5-year OSs were 70% (141/201) and 67% (118/176), respectively [52], suggesting that both IFN plus low-dose MTX and IFN plus retinoid are effective and well-tolerated in patients with refractory/relapsed CTCL [52].

IFN-γ is another IFN that is clinically available for the treatment of MF in Japan [60]. The ORR was 60% (9/15) as estimated by mSWAT, and the median duration of stable disease, though not reached, was ≥170 days (range, 29 to ≥253 days) [59]. The most-common treatment-related AE was flu-like symptoms in all patients (100%). The incidence of SAEs was 13.3% (2/15), including one case each of aggravation of MF and aggravation of cataract.

#### 3.2.2. Bexarotene

Bexarotene is a third-generation retinoid X receptor (RXR)-selective retinoid that has been approved by the European Medicines Agency for use in the treatment of both early and advanced CTCL [60,61]. Since bexarotene has been used for decades in the treatment of CTCLs, several preclinical studies have suggested the anti-CTCL mechanisms of bexarotene both in vitro and in vivo [12,15,61]. Notably, multiple reports have suggested the significance of chemokine production and chemokine receptor expression in the lesional skin of CTCL [12,15]. For example, bexarotene reduces the expression of CCR4 in CTCL cells in vitro, suggesting that bexarotene might inhibit the migration of CCR4-expressing CTCL cells in the lesional skin of patients with CTCL [15]. More recently, Tanita et al. reported that bexarotene reduces CCL22 production by M2 macrophages, leading to decreased serum CCL22 levels in patients with CTCL [12]. Given that CCL22 recruits CCR4+ CTCL cells and that a substantial number of M2-polarized TAMs are distributed in the lesional skin of patients with CTCL [4,47], those researchers concluded that bexarotene may inhibit the recruitment of CCR4+ CTCL cells, thereby suppressing CTCL disease activity [12]. Other in vitro experiments indicate that bexarotene not only suppresses the chemotaxis of CTCL cells but also selectively increases integrin β7 expression in CTCL cells, leading to growth arrest and apoptosis [11]. Together, these reports suggest the mechanism of bexarotene’s anti-tumor efficacy in patients with CTCL.

Indeed, several clinical trials have confirmed the anti-CTCL effects of bexarotene [60]. Duvic et al. reported the results of a multinational phase II-III trial to evaluate the efficacy and safety profiles of bexarotene for the treatment of refractory advanced-stage CTCLs [60]. The ORR was 45% (25/56) for patients dosed at 300 mg/m^2^/days and 55% (21/38) for patients dosed at >300 mg/m^2^/days, and the median DOR was 299 days [60]. The most frequent drug-related AEs were hypertriglyceridemia, hypercholesterolemia, hypothyroidism, and headache [60]. In a Japanese population, the ORR at 24 weeks was 61.5% (8/13) as assessed using mSWAT in a phase I/II clinical trial [62], and 65.5% (19/29) in a multi-center retrospective study [63]. The most frequent drug-related AEs were hypothyroidism (93.8–96.6%), hyperlipidemia (81.3–93.1%), and leukopenia (31.0–68.8%) [11,62]. The incidences of SAEs ranged from 20.7 to 25.0% [54,63]. Overall, bexarotene is effective and well-tolerated for the treatment of patients with early- and advanced-stage CTCLs [54,60,62,63].

### 3.3. HDAC Inhibitors (Vorinostat, Romidepsin, Quisinostat)

HDAC inhibition restores histone acetylation in CTCL cells to normal levels, thereby activating gene expression and leading to the induction of growth arrest, cellular differentiation, and apoptosis [64,65]. Indeed, various HDAC inhibitors have been investigated for the treatment of CTCL. Among these compounds, vorinostat has been approved by the FDA for the treatment of advanced CTCL. Initial clinical trial reports found an ORR of 29.5% for patients with CTCL at Stage IIB or higher [64]. Median time to progression was 9.8 months for responders at Stage IIB or higher. The most common drug-related AEs were diarrhea (49%), fatigue (46%), nausea (43%), and anorexia (26%), and the most common SAEs were fatigue (5%), pulmonary embolism (5%), thrombocytopenia (5%), and nausea (4%). Notably, in another clinical trial, the median PFS with vorinostat was 3.1 months (95% CI: 2.9–4.1 months) for relapsed CTCL, which is a value that is inferior to that obtained with mogamulizumab (HR 0.53; 95% CI 0.41–0.69; stratified log-rank *p* < 0.0001) [26].

Romidepsin is an another potent HDAC inhibitor that is isolated from the bacterium *Chromobacterium violaceum*; this compound has been approved by the FDA for the treatment of CTCL and peripheral T cell lymphoma (PTCL) [65]. The first phase II multi-institutional trial of romidepsin monotherapy for CTCL showed an ORR of 33.8% (95% CI: 23–46%) with four cases of CR (5.6%) and 20 cases of PR (28.2%), and the median DOR was 13.7 months. Safety profiles included nausea (73.2%), fatigue (57.7%), vomiting (26.8%), transient thrombocytopenia (56.3%), granulocytopenia (50.7%), anemia (52.1%), and leukopenia (42.3%).

Quisinostat is a second-generation pan-HDAC inhibitor with a broad spectrum of preclinical anti-tumor activity against various hematological malignancies, including CTCL [66]. Recently, the results of a phase II multicenter trial of oral quisinostat in 26 patients with previously treated MF or SS were reported [66]. The ORR was 24% as assessed by cases with >50% reduction of mSWAT score; the DOR in skin ranged from 2.8 to 6.9 months. The most common drug-related AEs were nausea, diarrhea (23%), asthenia (15%), hypertension (8%), thrombocytopenia (11%), and vomiting (11%) [66]. The incidence of SAEs was 11.5%, including single cases of hypertension, lethargy, and pruritus. Overall, quisinostat appears to be better tolerated than first-generation HDAC inhibitors such as vorinostat or romidepsin.

### 3.4. Anti-Metabolic Drugs: Pralatrexate, Methotrexate (MTX)

Paralatrexate is an antineoplastic folate analog similar to MTX; both compounds exhibit high affinity for the reduced folate carrier type-1 oncoprotein [67,68,69]. Paralatrexate inhibits dihydrofolate reductase, thereby disrupting DNA synthesis and leading to the induction of cytotoxicity against CTCL cells [67]. In a preclinical study, paralatrexate showed superior activity against human lymphoma cells compared to MTX [68]. A phase I dose de-escalation study for relapsed or refractory CTCL suggested that the ORR of pralatrexate was 45% (13/29) (95% CI: 26.4–64.3%) with 1 case of CR and 12 of PR [67]. The most common AEs were mucositis (48%), fatigue (41%), nausea (31%), edema (28%), epistaxia (21%), pyrexia (21%), anorexia (21%), and skin toxicity (21%). The most common SAEs were mucositis (17%) and leukopenia (3%). More recently, Duvic et al. reported the results of a phase I/II open-label, multicenter clinical trial for pralatrexate (15 mg/m^2^) plus bexarotene (150 mg/m^2^) combination therapy for relapsed or refractory CTCL [70]. The ORR was 60% (18/31), including four cases with CR and 14 with PR; the DOR for CR patients was 9.0 to 28.3 months. The median PFS was 12.8 months [69]. The most common AEs with this combination therapy were stomatitis (65%), hypertriglyceridemia (56%), fatigue (44%), nausea (32%), neutropenia (32%), central hypothyroidism (24%), and anemia (24%) [70]. The most common SAEs were neutropenia (35%), hypertriglyceridemia (29%), and stomatitis (21%) [69] Overall, pralatrexate is effective for relapsed or refractory CTCL with acceptable toxicity, especially when administered in combination with bexarotene.

MTX is an analog of folic acid that has been used classically (since 1964) for the treatment of MF [70,71]. In addition, a retrospective study of low-dose MTX for the treatment of patients with MF has been conducted [71]. The ORR of low-dose MTX for MF was 30.4% (21/69), including seven cases with CR and 14 with PR [71]. Notably, most of the responding patients had early-stage (patch or plaque) MF, with the exception of one patient with tumor-stage disease [71]. In addition, the results of a multicenter observational study of MTX for the treatment of patients with MF found an ORR of 70.9% (56/79) [70]. Those authors concluded that the response of subjects with MF to MTX depended on the dose of MTX and the stage of MF [70]. Overall, low-dose MTX monotherapy is well-tolerated and effective, especially for MF, notably in early-stage disease.

### 3.5. Miscellaneous Therapies Preferred Systemic Therapies: Gemcitabine, Pegylated Liposomal Doxorubicin, and Extracorporeal Photopheresis (ECP)

As described above, the NCCN guidelines for primary cutaneous lymphomas suggest a systemic therapy option for the treatment of advanced MF/SS [1]. Among cytotoxic drugs for CTCL, gemcitabine and pegylated liposomal doxorubicin are recommended as SYST-CAT B together with brentuximab vedotin and pralatrexate [1]. Gemcitabine is a pyrimidine antimetabolite that has been used for the treatment of advanced CTCL. Two phase II studies have been reported. The first found an ORR of gemcitabine monotherapy of 75% (24/32) for CTCL (including MF, peripheral T cell lymphoma, and SS), with seven cases of CR, and 73% (19/26) for MF with six cases of CR; the median PFS for CTCL was 10 months [72]. In the second report, the ORR of gemcitabine monotherapy was 68% (17/25) for CTCL, with three cases of CR, and the median PFS for CTCL was 4.1 months [73]. Pegylated liposomal doxorubicin, which is doxorubicin encapsulated in liposomes that leads to decreased cardiotoxicity and nephrotoxicity, is recommended for the treatment of CTCLs in the NCCN guideline as SYST-CAT B [1]. A prospective multicenter study found an ORR of pegylated liposomal doxorubicin for MF/SS of 56% (14/25), with five CR (20%), and a median PFS of 5 months [74]. Although CHOP is the standard regimen in patients with non-Hodgkin’s lymphoma including CTCL [75], the DOR is limited compared to surface molecular-targeted therapy such as brentuximab vedotin [76]. Overall, these cytotoxic drugs could be other options for the treatment of CTCL, although the incident ratio of SAEs was high (36–40%) [74,75]. Etoposide, chlorambucil, cyclophosphamide, pentostatin, temozolomide, and bortezomib are also recommended in the NCCN guidelines as useful drugs under certain circumstances [1].

ECP is a leukapheresis-based therapy that exposes the isolated lymphocytes from peripheral blood to 8-methoxypsoralen and ultraviolet A radiation, and it has been used for the treatment of CTCL as SYST-CAT A [1,77]. The ORR of ECP for SS has been found to be 55.7% with a CR rate of 17.6% [78]. Notably, since ECP can be combined with other systemic therapies such as IFNs and bexarotene [79], ECP should be considered for the treatment of CTCL, although the availability of ECP might be limited.

## 4. Conclusions

As described above, various therapeutic options for CTCL have been reported, but the recommended therapeutic line remains under discussion (Table 1 and Table 2) [1,2]. MF, the most common of the cutaneous T-cell lymphomas (CTCLs), starts as an indolent disease, progressing from a patch stage to confluent plaques (early stage). However, among some CTCL patients, it subsequently develops into skin tumors (advanced stage), followed by lymph node and visceral involvement.

For the treatment of advanced CTCL, although the guidelines suggest three categories of systemic therapy options for the treatment of advanced MF/SS, the recommended first-line treatments in each category have yet to be established [1]. Moreover, since CTCL patients simultaneously possess different tumor stages (patch, plaque, and tumors), most systemic therapies are combined with local treatments. For example, since CCR4 is expressed not only on CTCL cells but also on regulatory T-cells [80], mogamulizumab might enhance the anti-tumor immune response at the tumor site, and it might be suitable for combination with local immunotherapies such as topical bexarotene or imiquimod. Brentuximab vedotin kills CD30+ CD163+ TAMs and might suppress tumor formation in MF [3], suggesting that brentuximab vedotin might be suitable for combination with topical steroids or phototherapy. Since patients with advanced-stage MF generally possess patch, plaque, and tumor lesions at the same time, the development of combination therapies using topical formulations and systemic therapies (for example, by combining immunomodulatory and immune cell-targeted therapies) will need to be investigated in future experiments. In the future, such combination therapies could be the recommended first-line treatments for advanced CTCL.

## Figures and Tables

**Table 1:** The efficacy of topical formulations for treatment of anti-cutaneous T cell lymphoma.

Drugs	Stage of Enrolled Patients	ORR (%)	CR (%)	PR (%)	Most Common AEs
Topical bexarotene gel		63	21	42	irritation
Mechlorethamine hydrochloride gel	stage IA/B	46			
Topical carmustine	stage IA	98	86	12	irritation
Topical carmustine	stage IB	84	47	37	irritation
Topical 5% imiquimod	stage IA-IIB	80	45	35	irritation

ORR: overall response rate, CR: complete response, PR: partial response, AE: adverse event.

**Table 2:** The efficacy of systemic formulations for treatment of CTCLs.

Protocol	ORR (%)	CR (%)	PR (%)	PFS	Most Common AEs (%)	Most Common SAEs (%)
Mogamulizumab	28			7.7 months	infusion reaction (32%)	pyrexia (4%)
Brentuximab vedotin	56.3				neuropathy (50%)	neuropathy (5%)
Denileukin diftitox	44	10	34	>2 years	fatigue (12%)	capillary leak syndrome (2%)
Pembrolizumab	37.5	8.3	29.2			
Alemtuzumab	51.1	17.9	33.3	3.4 months		severe infectious AEs (62%)
High-dose IFN-a2a	29	4	25			
IFN-a2a plus PUVA	80.6	74.6	6	32 months		
IFN-a2b with PUVA	93	73	20	>2 years		
IFN-g	60			>170 days	flu-like illness (100%)	
Bexarotene	45				hypertriglyceridemia	hyperlipidemia
Vorinostat	29.5			9.8 months	diarrhea (49%)	thrombocytopenia (5%)
Romidepsin	33.8	5.6	28.2	13.7 months	nausea (73.2%)	
Quisinostat	24			2.8–6.9 months	nausea, diarrhea (23%)	hypertension (11.5%)
Pralatrexate	44.8	3.4	41.4		mucositis (48%)	mucositis (17%)
Gemcitabine	75	21.8	53.1	10 months		
Pegylated liposomal doxorubicin	56	20	36	5 months		

PFS: progress free survival, SAE: severe adverse event.

## Data Availability

Data sharing not applicable.
